# Analysis of experimental data of environmental cement prepared by fly ash of eggs shell and sand dune for reduction of carbon dioxid

**DOI:** 10.1016/j.dib.2020.105407

**Published:** 2020-03-12

**Authors:** Abdelghani Brahimi, Mourad Meghachou, Hicham Abbad, Abdelkader Rahmouni, Mohammed Belbachir, Fatima Zohra Zeggai, Bachari Khaldoun

**Affiliations:** a Department of Civil Engineering, Laboratory of Civil Engineering and Environment, Djillali Liabes University, 22000 Sidi Bel-Abbes, Algeria; b Department of Chemistry, Laboratory of Polymer Chemistry -University of Oran1 Ahmed Benbella. BPN°1524 El'Menouer, 31000 Oran, Algeria; c Centre de Recherche Scientifique et Technique en Analyses Physico-chimiques (CRAPC), BP 38Bou-Ismail-RP, 42004 Tipaza, Algeria

**Keywords:** Fly ash, Cement, Geopolymer, Sand dune, Sol-gel reaction, Eggs shell, Carbon dioxid, Environment

## Abstract

In the recent years, the dominant cementitious materials have been industrial by products such as fly ash. This present data describes some of the cementitious products that are attracting attention in the global research community and the properties and characteristics of these materials that affect their performance such durability, mechanically properties and reduction of carbon dioxid (CO_2_). The present investigation deals with the chemical synthesis of cementitious material using fly ash of eggs shell rich in calcium(Ca) and sand dune(southern west of Algeria) rich in silica(SiO_2_).The composition of geopolymers synthesized are the most compressive resistant with a maximum stress of 49.71 MPa, the most flexible (E = 2.63 GPa) and the most ductile (εr = 65.42%).The characteristic properties of the chemically synthesized cementitious materials were analyzed by the chemical composition analysis XRF, XRD and SEM analyses.

Specifications table

**Table:** 

Subject	Polymer chemistry, Chemical engineering, materials science, nanotechnology, civil engineering
Specific subject area	Polymer chemistry, Chemical engineering, materials science, nanotechnology, civil engineering
Type of data	Table, Image and Figure
How data were acquired	SEM, XRD, XRF
Data format	Raw and analyzed
Parameters for data collection	Four samples of new cement concrete prepared from sand dune (Algerian MMT) exchanged with fly ash of eggs shell via chemical reaction*. Material prepared was analyzed by their elemental composition as well as the morphological and crystalographic properties.* Parameters for the initial structures are provided in this article.
Description of data collection	Prepared cement concrete were used as new environmentally materials for construction especially for reduction of carbon dioxid (CO_2_).
Data source location	Republic Algerian democratic and popular
Data accessibility	Data are supplied with this article
Related research article	Ayat, M., Belbachir, M., Rahmouni, A*. (2018). Cationic polymerization of poly(α-methylstyrene-block-isobutyl vinyl ether) using Maghnite-H^+^ clay (Algerian MMT) as catalyst. *Polymer bulletin*, 75(12),5355-53-71.

## Value of the data

• The data in this article will be informative to synthesis of new environmentally simontinious materials based on sand dune and eggs shell as raw materials.
• By using these data, researchers can make comparisons with other portlandite cement.
• Strategy for this method of synthesis employed in this Data article can be used as a reference for future studies in the environment and construction domain.
• The Data obtained in this work can be effectively applied for the synthesis of simontinious material from raw materials such sand dune and fly ash of eggs shell.
• The data can be highlighted for further studies in development of better strategy for synthesis of geopolymeric materials especially for civil engineering domain.

## 1 Data

**Scheme 1:**
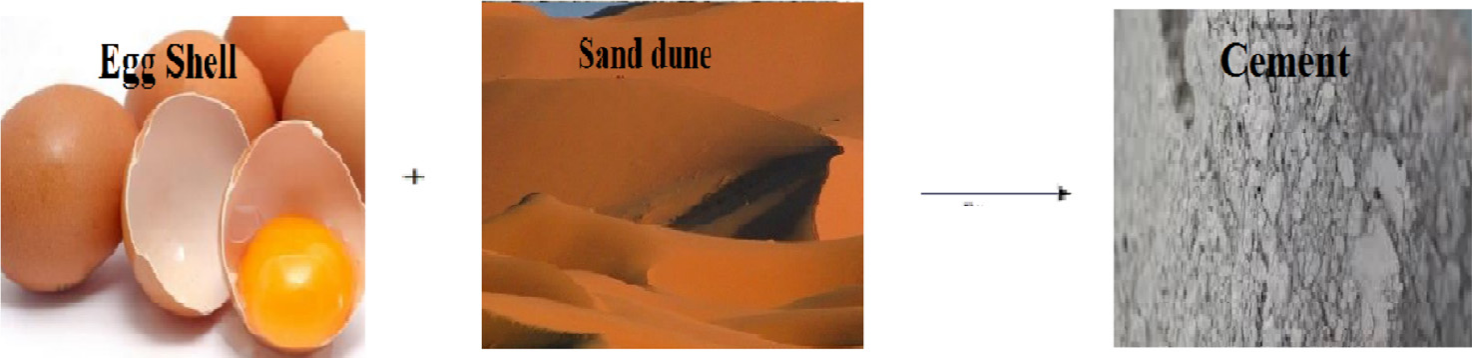
Describes a new design of simontinious materials consisting on sand dune and fly ash of eggs shell as raw materials for reduction of carbon dioxid.

**Fig. 1:**
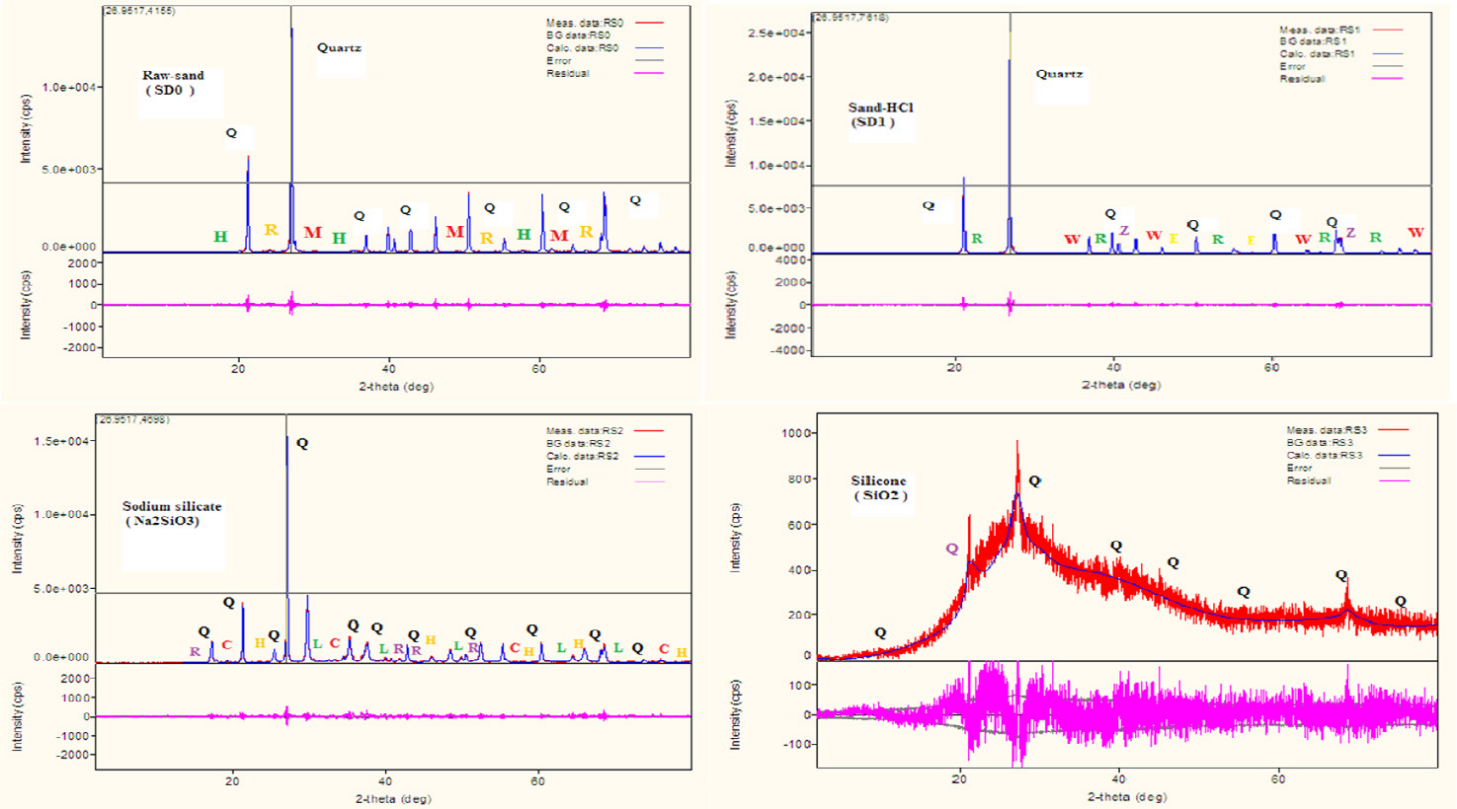
XRD patterns of raw sand, sand treated with 1 M HCl, sodium silicate Na_2_SiO_3_ and silicon dioxid SiO_2_.

**Fig. 2:**
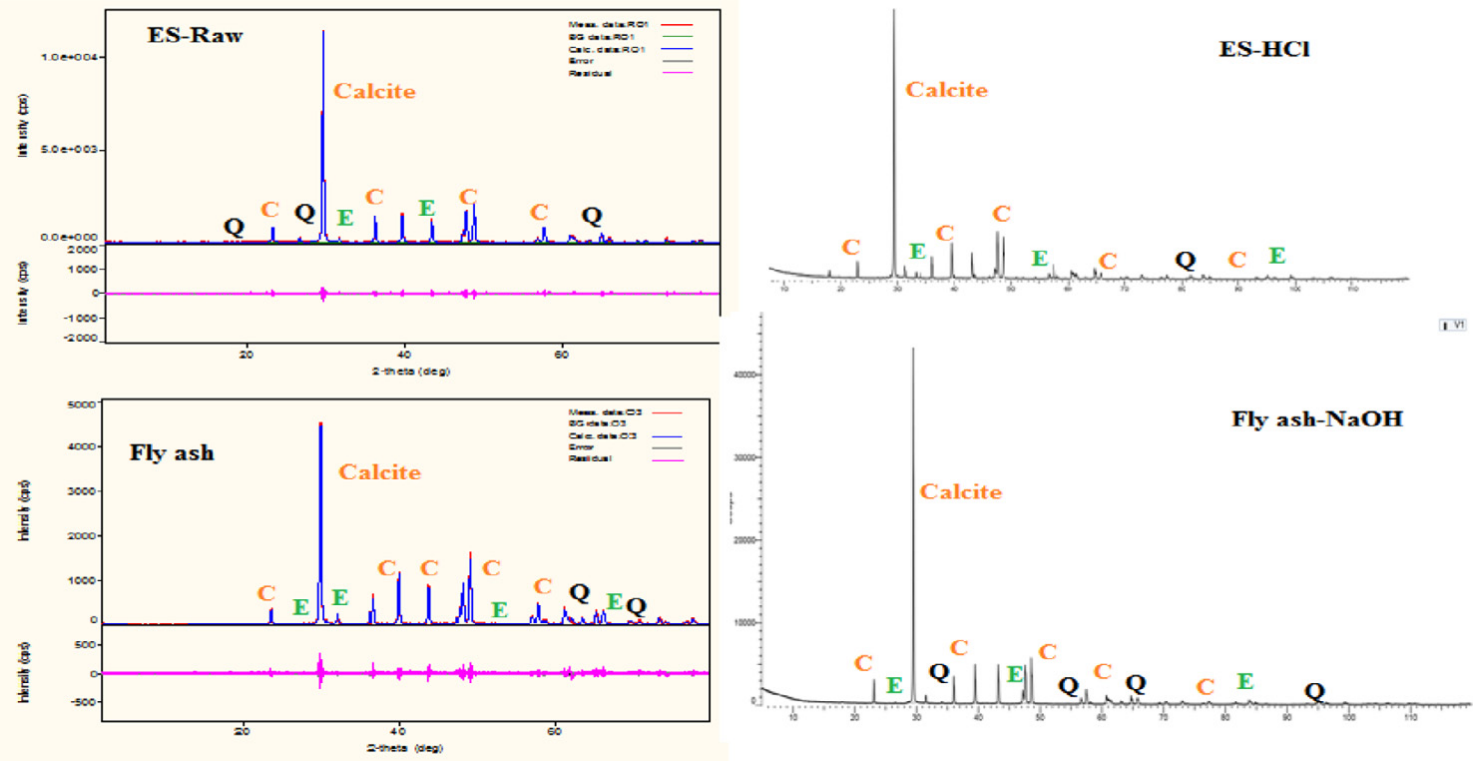
XRD patterns of different form of eggs shell and fly ash.

**Fig. 3:**
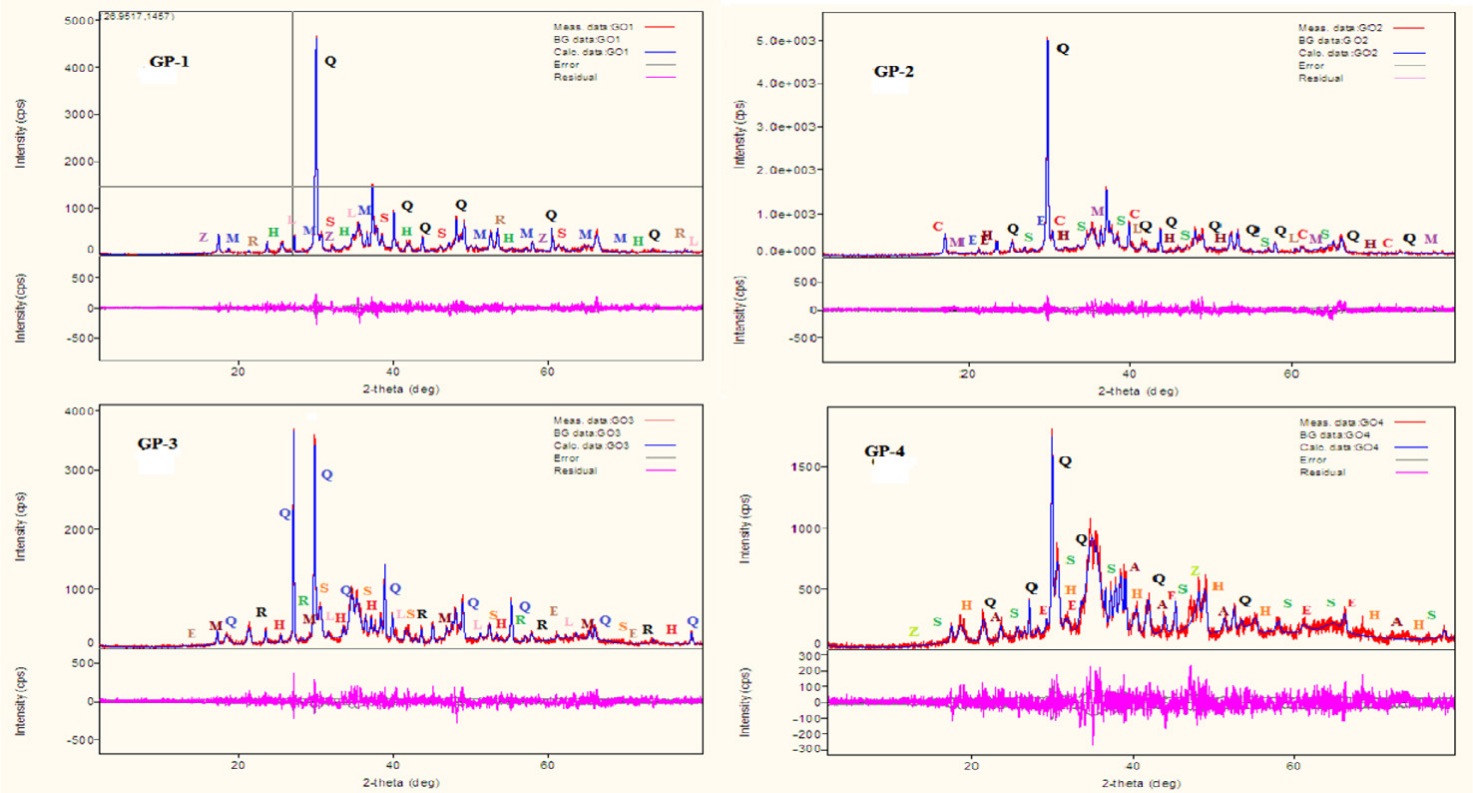
The XRD pattern of geopolymer (GP-1,GP-2,GP-3 and GP-4) with NaOH molar ratio variation of 13 M.

**Fig. 4:**
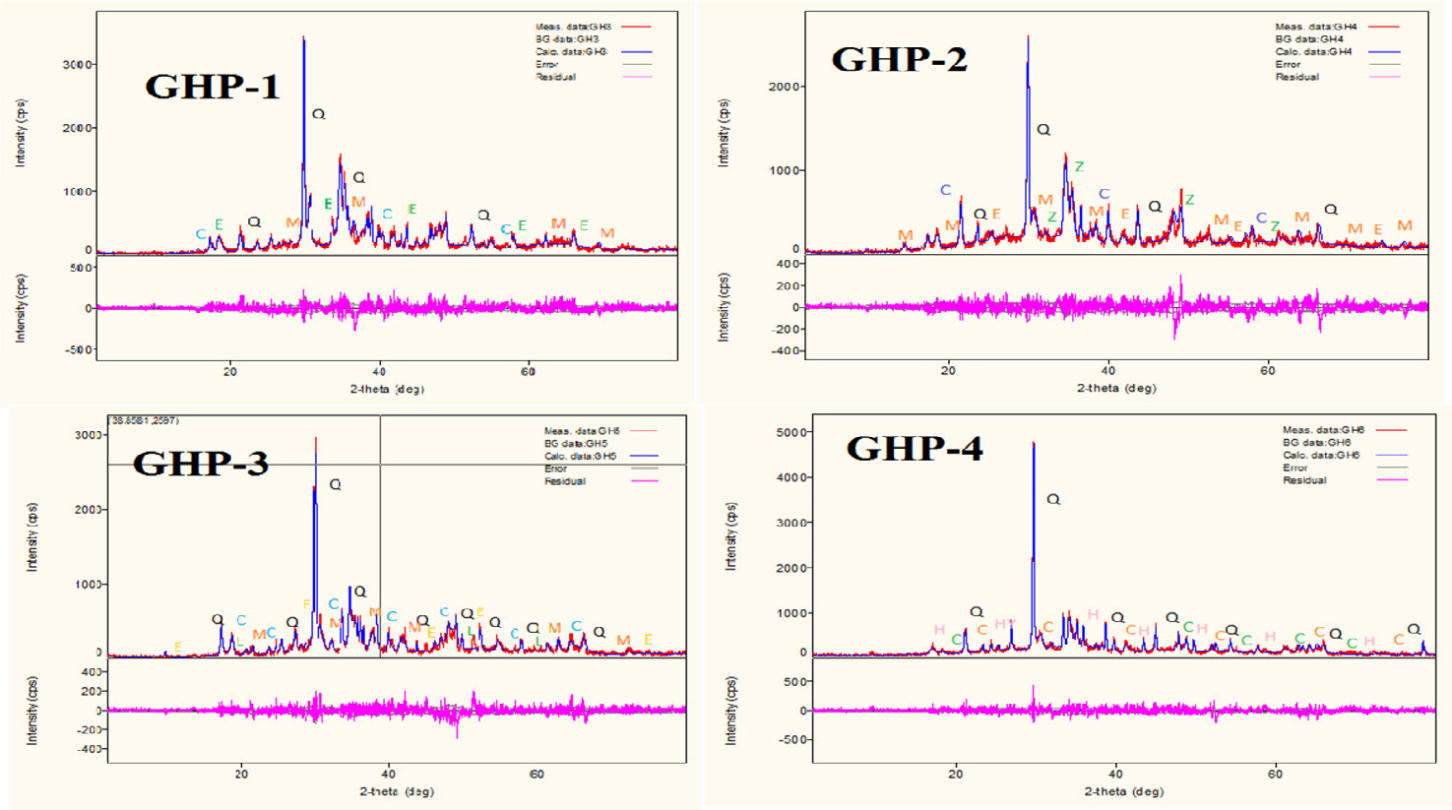
The XRD pattern of hybrid geopolymer (GHP-1,GHP-2,GHP-3 and GHP-4) with NaOH molar ratio variation of 13 M.

**Fig. 5:**
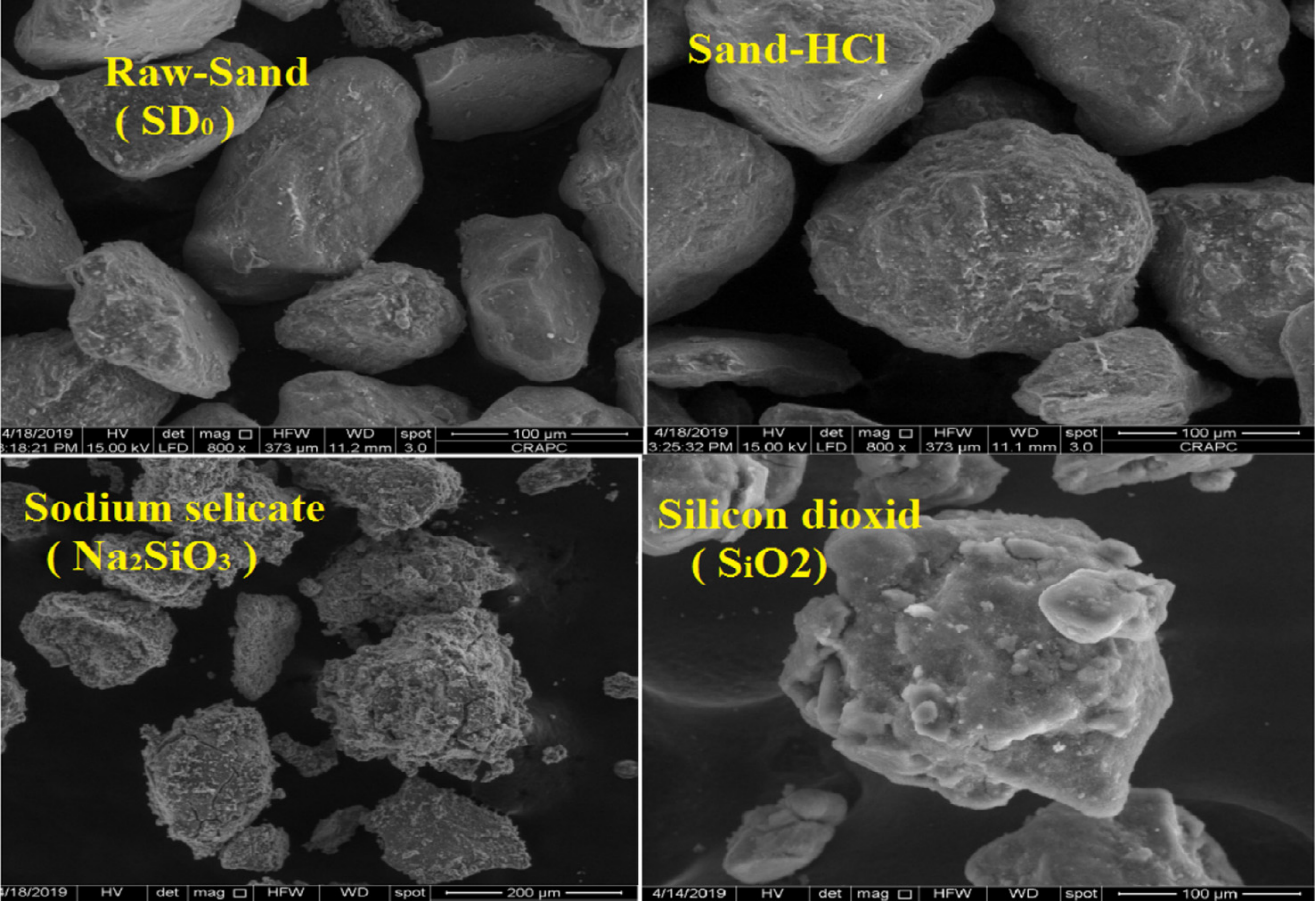
SEM micrographs of the different forms of sand dune (southern of Algeria).

**Fig. 6:**
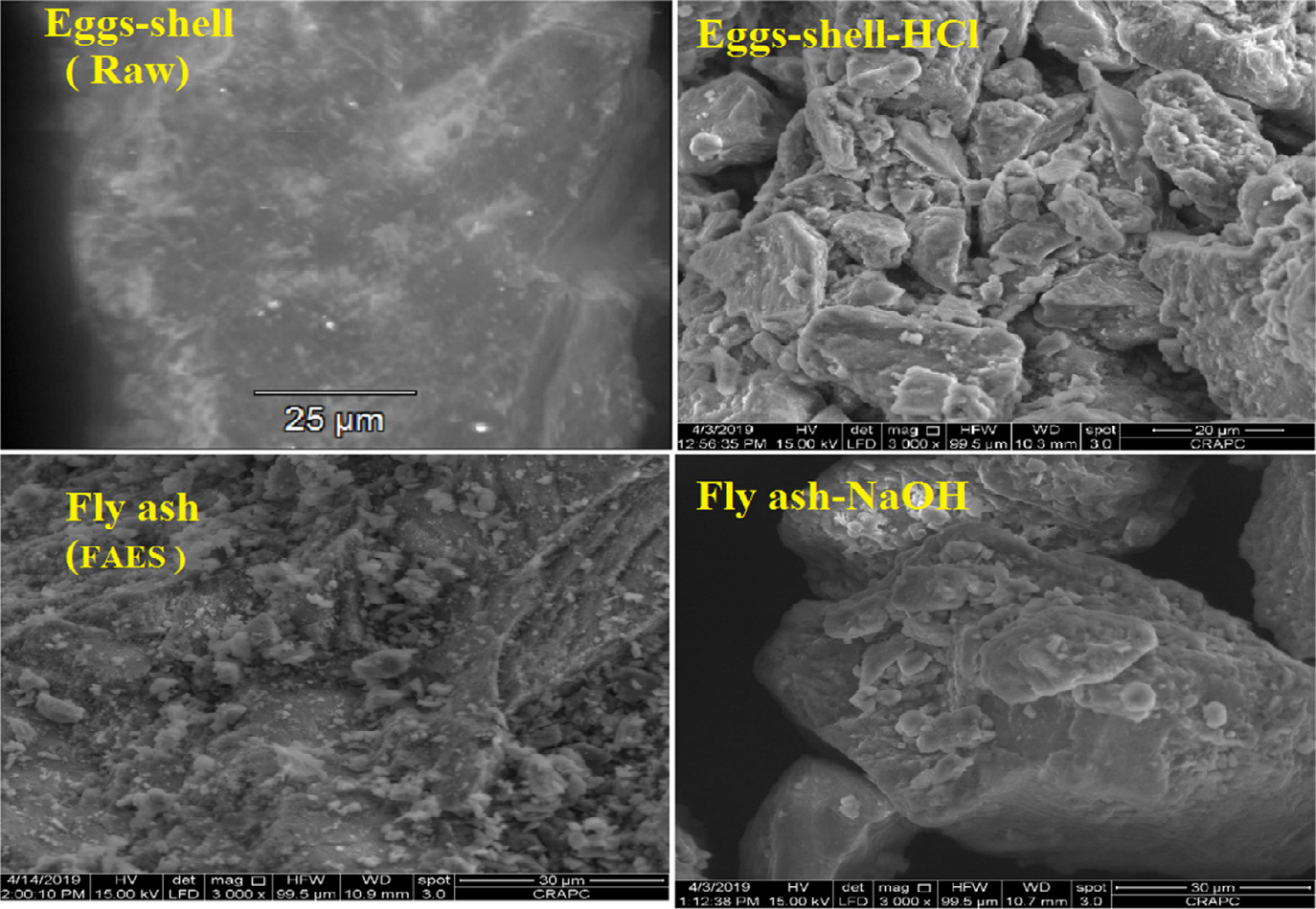
SEM micrographs of the different forms of eggs shell and fly ash egg shell (FAES: Fly ash eggs shell).

**Fig. 7:**
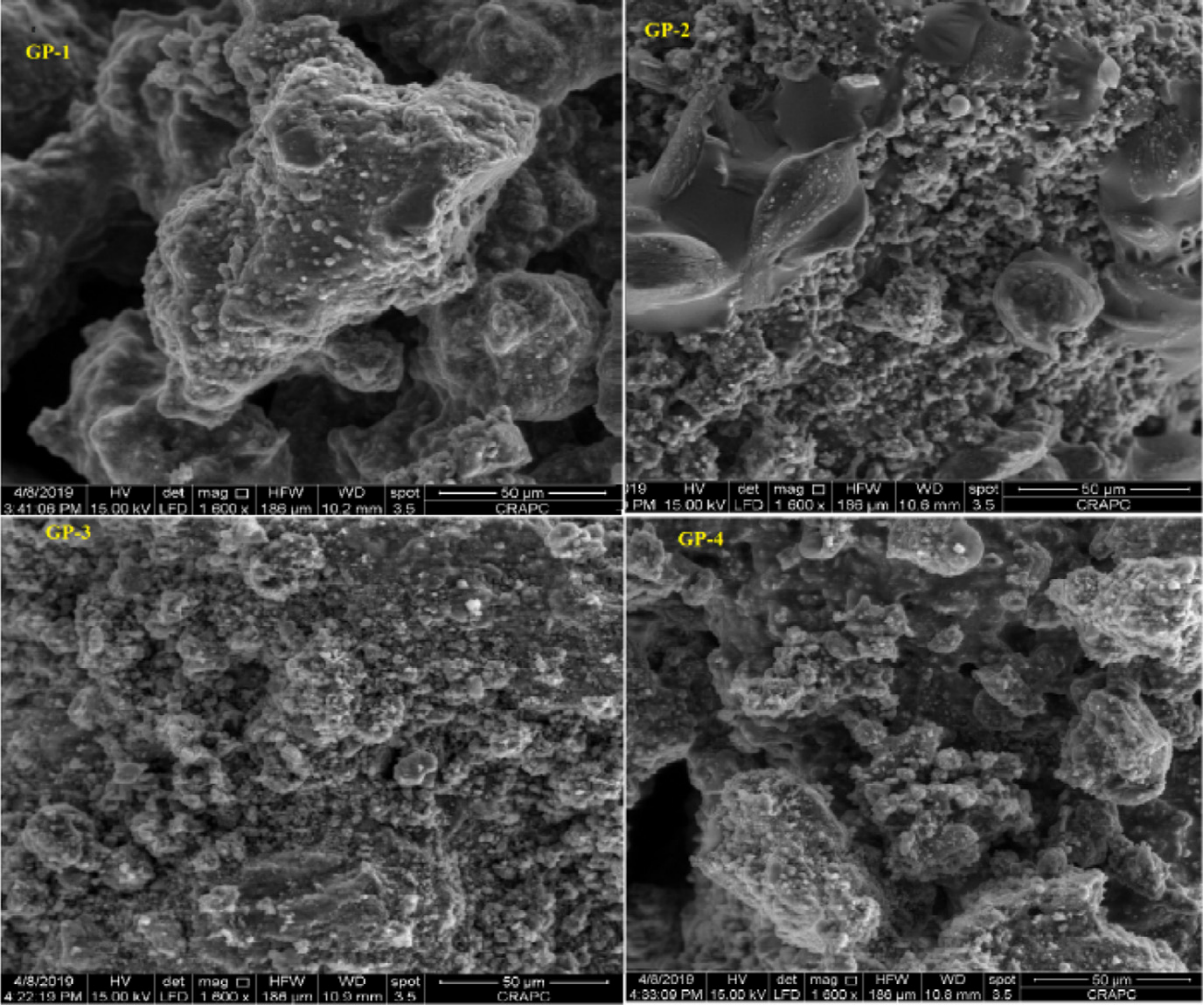
SEM micrographs of the foamed geopolymer blocks vs. fly ash egg shell and sand dune (FAES: Fly ash, GP-1, GP-2, GP-3, GP-4: 1 h).

**Fig. 8:**
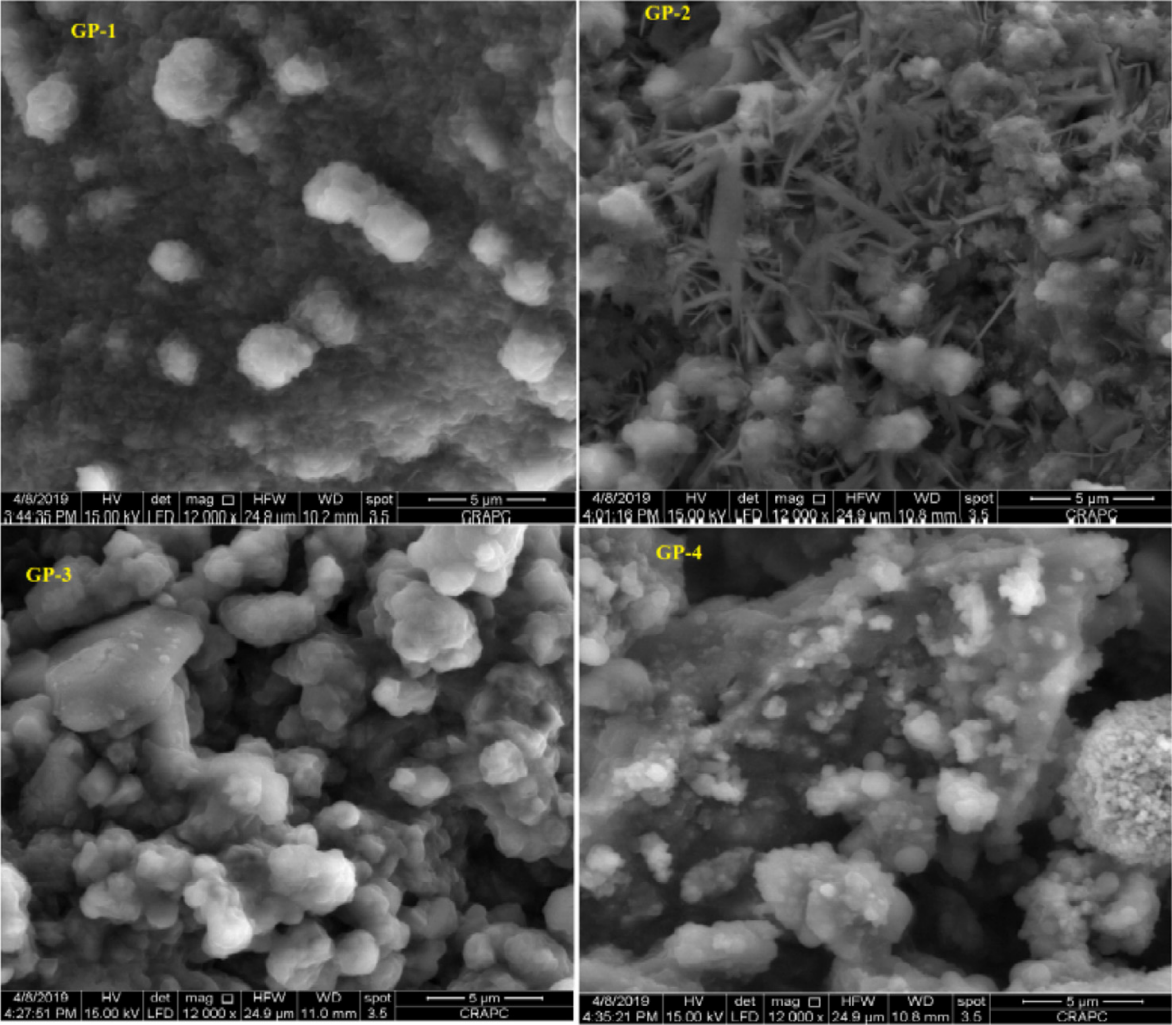
SEM micrographs of the foamed geopolymer blocks vs. fly ash egg shell and sand dune(FAES: Fly ash, GP-1, GP-2, GP-3, GP-4: 24 h).

**Fig. 9:**
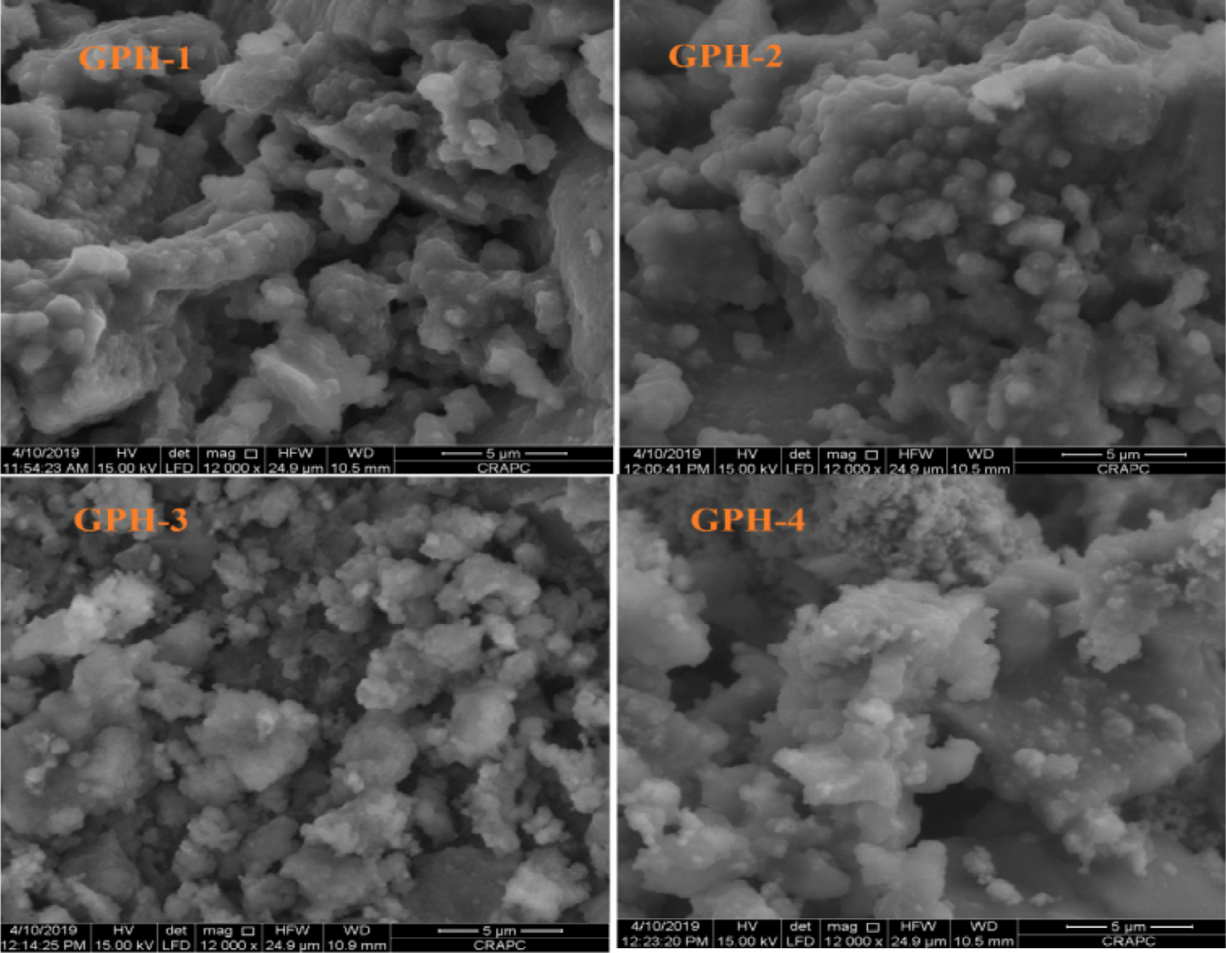
SEM micrographs of the foamed hybrid geopolymer blocks vs. fly ash egg shell (FAES: Fly ash, GHP-1, GHP-2, GHP-3, GHP-4: 1 h).

**Fig. 10:**
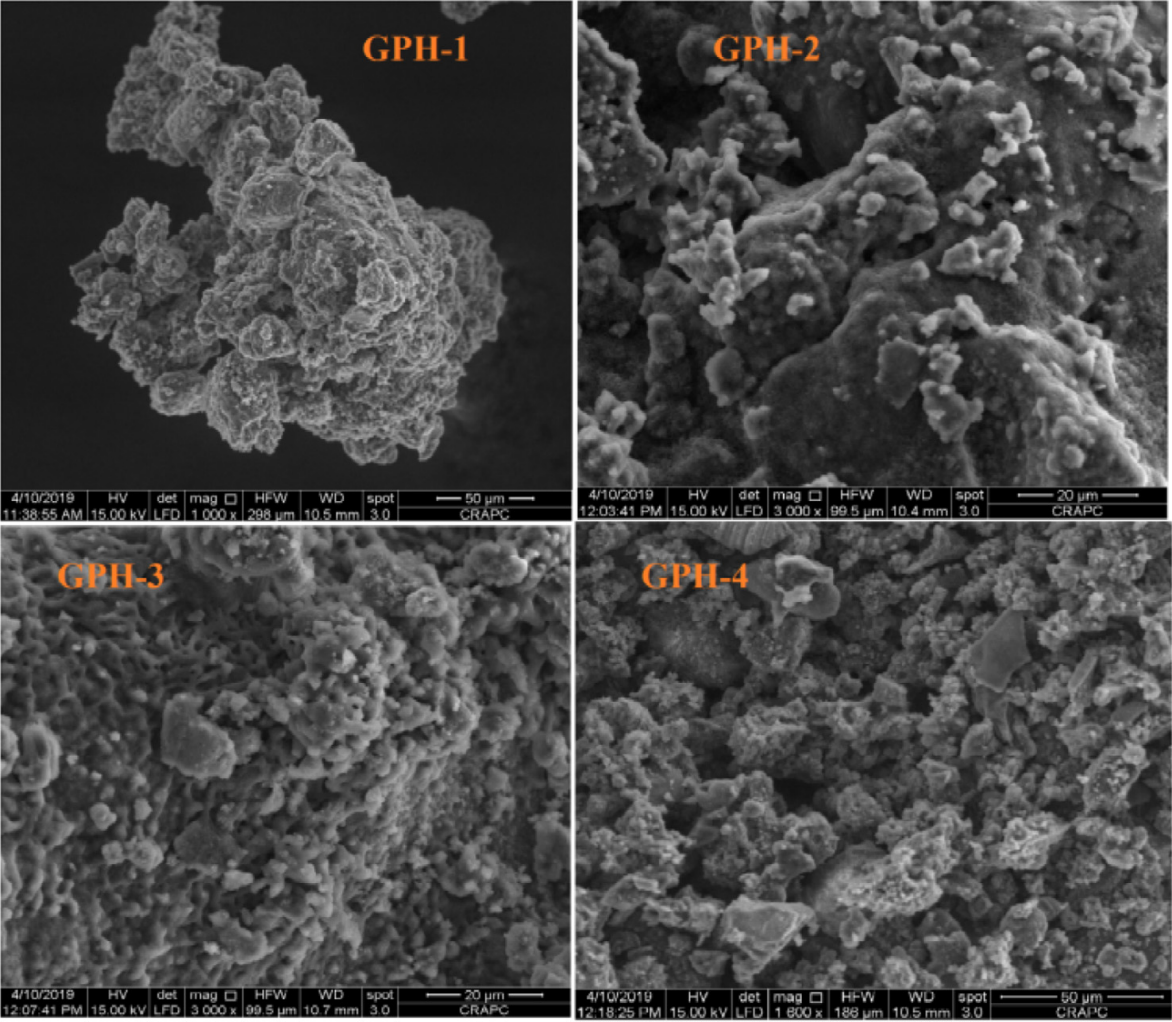
SEM micrographs of the foamed hybrid geopolymer blocks vs. fly ash egg shell (FAES: Fly ash, GHP-1, GHP-2, GHP-3, GHP-4: 24 h).

The data described in this paper provides new design for synthesis of new environmentally simontinious material based on raw material sand dune and fly ash of eggs shell used in construction domain. The formation of geopolymers and hybrid geopolymers was confirmed by XRF, XRD, and SEM [1]. Scheme 1 describes a new design of simontinious materials consisting on sand dune and fly ash of eggs shell as raw materials. Table 1 describes chemical composition of the raw sand dune (southern west of Algeria). Table 2 describes chemical composition of the differents form of sand dune. Table 3 describes **c**hemical composition of the fresh fly ash of egg shell (FAES). Table 4 describes chemical composition of fly ash, fly ash actived and different form of eggs shell (ES). Table 5 summarizes chemical procedure for synthesized geopolymers materials (GPs). Table 6 summarizes chemical procedure for synthesized geopolymers materials (GHPs). Table 7 describes Mechanical properties of fly ash, sand dune and geopolymers samples. Table 8 summarizes Tension strength of different geopolymers synthesized (GPs). Figs. 1 and 2 describe XRD patterns of raw sand, sand treated with 1 M HCl, sodium silicate Na_2_SiO_3_ and silicon dioxid SiO_2_ and XRD patterns of different form of eggs shell and fly ash. Fig. 3 describes the XRD pattern of geopolymers (GP-1,GP-2,GP-3 and GP-4) with NaOH molar ratio variation of 13 M. Fig. 4 describes the XRD pattern of hybrid geopolymer (GHP-1,GHP-2,GHP-3 and GHP-4) with NaOH molar ratio variation of 13 M. Figs. 5 and 6 describe SEM micrographs of the different forms of sand dune (southern of Algeria) and SEM micrographs of the different forms of eggs shell and fly ash egg shell (FAES: Fly ash eggs shell). Figs. 7 and 8 describe SEM micrographs of the foamed geopolymer blocks vs. fly ash egg shell and sand dune (FAES: Fly ash, GP-1, GP-2, GP-3, GP-4: 1 h and 24 h). Figs. 9 and 10 SEM micrographs of the foamed hybrid geopolymer blocks vs. fly ash egg shell (FAES: Fly ash, GHP-1, GHP-2, GHP-3, GHP-4: 1 h and 24 h).

**Table 1:** Chemical composition of the raw sand dune (Western of Algeria).

Oxide	Content %	Oxide	Content %
SiO_2_	90.83	Na_2_O	0.01
Al_2_O_3_	7.45	CaO	1.02
Fe_2_O_3_	0.29	MgO	0.00
K_2_O	0.01	TiO_2_	0.09

**Table 2:** Chemical composition of different form of Sand dune (SD).

Samples				
Oxides (w%)	SD-Raw	SD-HCl	Water glass (Na_2_SiO_3_)	Silicone (Si)
SiO2	90.04	90.83	96.99	99.16
Al2O3	13.56	12.45	9.61	0.65
Fe2O3	0.59	0.29	0.29	0.28
CaO	1.06	1.02	0.21	0.18
MgO	0.00	0.00	0.00	0.00
SO3	0.35	0.32	0.65	0.59
K2O	0.00	0.01	0.00	0.63
Na2O	0.02	0.01	0.00	0.00
P2O5	0.06	0.09	0.00	0.09
TiO2	0.12	0.09	0.04	0.08
Cr2O3	0.00	0.00	0.00	0.00
Mn2O3	0.00	0.00	0.00	0.00
ZnO	0.00	0.00	0.00	0.00
SrO	0.02	0.01	0.01	0.01
CO2	0.00	0.00	0.00	0.00
LOI

**Table 3:** Chemical composition of the fresh fly ash of egg shell (FAES).

Oxide	Content %	Oxide	Content %
SiO_2_	0.06	Na_2_O	2.92
Al_2_O_3_	0.04	CaO	63.69
Fe_2_O_3_	0.01	MgO	0.57
K_2_O	0.04	TiO_2_	0.02

**Table 4:** Chemical composition of fly ash, fly ash actived and different form of eggs shell (ES).

Samples				
Oxides (w%)	ES-Raw	ES-HCl	Fly ash	Fly Ash-NaOH
SiO2	0.240	0.060	0.002	0.060
Al2O3	- - -	0.040	- - -	0.030
Fe2O3	0.040	0.010	0.007	0.010
CaO	61.130	35.390	63.69	36.230
MgO	0.500	0.520	0.570	0.530
SO3	0.100	0.090	0.090	0.090
K2O	0.042	0.040	0.060	2.950
Na2O	0.085	2.920	0.190	0.210
P2O5	0.020	0.250	0.280	0.230
TiO2	0.017	- - -	0.020	- - -
Cr2O3	0.006	- - -	0.010	- - -
Mn2O3	0.006	- - -	0.010	- - -
ZnO	0.005	0.004	0.010	0.010
SrO	0.025	0.012	0.030	0.010
CO2	- - -	59.800	- - -	58.640
LOI	38.600	60.650	34.950	59.650

**Table 5:** Procedure for synthesized geopolymers (GPs).

Geopolymers (GPs)	Mass (g) ratio
GP-1: Na2SiO3, FAES, NaOH,H2O	1.75: 3.00: 1.00: 2.25
GP-2: Na_2_SiO_3_, FAES, NaOH, SiO_2_, H2O	1.75: 3.00: 1.00: 0.5: 2.25
GP-3: Na_2_SiO_3_, FAES, NaOH, Al_2_O_3_, H_2_O	1.75: 3.00: 1.00: 0.5: 2.25
GP-4: Na_2_SiO_3_, FAES, NaOH, Fe_2_O_3_, H_2_O	1.75: 3.00: 1.00: 0.5: 2.25

**Table 6:** Procedure for synthesized geopolymers hybrid (GHPs).

Hybrid geopolymers (GHPs)	Mass (g) ratio
GHP-1: Na2SiO3, FAES, NaOH, H2O, PET	1.75: 3.00: 1.00: 2.25: 0.5
GHP-2: Na_2_SiO_3_, FAES, NaOH, SiO_2_, H2O,PET	1.75: 3.00: 1.00: 0.5: 2.25: 0.5
GHP-3: Na_2_SiO_3_, FAES, NaOH, Al_2_O_3_, H_2_O,PET	1.75: 3.00: 1.00: 0.5: 2.25: 0.5
GHP-4: Na_2_SiO_3_, FAES, NaOH, Fe_2_O_3_, H_2_O,PET	1.75: 3.00: 1.00: 0.5: 2.25: 0.5

**Table 7:** Mechanical properties of fly ash, sand dune and geopolymers samples.

Sample	Fly ash (w %)	Young's modulus (GPa)	Deformation at break (%)	Maximum stress (MPa)	Yield strength (MPa)
Sand dune	–	0.86	20.09	15.33	–
Fly ash	–	1.43	37.95	31.06	55.25
Fly ash-NaOH	–	1.72	42.01	39.82	70.94
GP-1	5	1.75	45.21	41.09	70.66
GP-2	10	1.96	49.97	49.71	80.94
GP-3	20	1.85	49.61	47.45	80.83
GP-4	30	1.73	48.05	45.59	79.19
GP-5	40	1.71	47.43	42.36	78.10
GP-7	50	1.52	45.31	40.24	75.06

**Table 8:** Tension strength of different geopolymers synthesized (GPs).

Geopolymers	Tension at 28 days (kN/mm²)
GP-1	1.88
GP-2	2.1
GP-3	1.74
GP-4	1.59

## 2 Experimental design, materials, and methods

### 2.1 Chemical and material

All reagents in this work were of analytical grade and used as received without further purification. NaOH and HCl (36%) were used as initiator from sigma Aldrich (French). The sand dune used in this work came from a quarry located in Naama (southern west of Algeria).

### 2.2 Preparation and treatment of sand dune

The silica sand in these experiments was from dune sand (west south of Algeria). For the first part of the experiment, 100 g of dry sand have been processed by 200 ml of hydrochloric acid for 30 minutes at ambient temperature. For the second part of experiment, all leaching tests were conducted in a 250 ml glass beaker placed on a heater, with a magnetic stirrer having a controller unit. When the required temperature (80 °C) of the beaker contents (100 ml of acid) was reached, approximately 30 g of dry sand were added into the beaker, while the contents of the beaker has been stirred at a constant speed of 200 rpm. The beaker was covered to prevent losses by evaporation. From leaching solution an amount of sample of the reaction mixture was taken out at pre-determined time intervals, filtered, repeatedly washed with distilled water to remove any unspent acid and then dried at 110 °C for one hour. The experimental parameters are presented in Tables 1 and 2.

### 2.3 Preparation of fly ash

A fly ash sample was collected from a eggs shell as raw material washed firstly with distilled water and then by acidic solution (HCl 1 M) to remove impurity and minimize rate of limine(CaO). After drying and when all moister was removed then this sample takes for calcination under 700–850 °C then dried at 25 °C. The XRF analysis was conducted to characterize the fly ash sample. The XRF test is shown in Tables 3 and 4.

### 2.4 Synthesis of sodium silicate

The sodium silicate called water glass (Na_2_SiO_3_) was synthesized in our laboratory (laboratory of polymer chemistry at oran1 university -Algeria) by using 100 g of sand dune washed with acidic solution (HCl 1 M) and dried at 25 °C then mixed with 200 g of sodium hydroxid (NaOH 13 M). The mixture was fused using a platinum crucible using in an electrical-fired furnace at 850 °C for one hours and a heating rate of 5 °C/min. The melt was left to cool and solidify in the crucible. Our procedure managed to synthesize 70 g of sodium silicate nanomaterials (water glass), it is white powder, then was dried at 25 °C for microstructural, chemical and mineralogical analysis.

### 2.5 Synthesis of geopolymers and hybrid geopolymers

The geopolymer and hybrid geopolymer concrete was prepared by conventional method [2]. Four mixes were made. Four mixes (GP1, GP2, GP3, and GP4) of geopolymer and four hybrid geopolymer (GHP1, GHP2, GHP3 and GHP4) concretes using NaOH (13 M) were prepared as show in Tables 5 and 6. The synthesis of the alternative cementitious material using the hydrothermal process was performed subsequent to the preparation of the silica and alumina source materials [3]. This work describes the valorization of raw and actived fly ash of eggs shell and Algerian sand dune for preparation of geopolymers mortars (cementitious materials) by alkaline activation. Sodium silicate (Na_2_SiO_3_) was prepared and characterized in our laboratory. The chemical composition by XRF indicate that sand dune (southern of Algeria) deposit has a high concentration of quartz (90.04. to 99.16% silica) with low concentration of others oxides. The microscopic observations reveals several morphologies of sand, some are elongated, rounded and angular with presence of pores [4]. The fly ash of eggs shell exhibit better performance than ordinary cements on water penetration, very good fire resistance and minimize carbon dioxid (CO_2_). The micro structure and the SEM results reveal that the egg shell is properly and evenly distributed in the matrix phase and has a good bonding between the egg shell particles and sand dune rich in silicium and aluminum [5]. The geopolymers and hybrid geopolymers resulting from the alkaline activation of eggs shell fly ash exhibit an amorphous character in general and it is determined that fly ash can be successively used with sand dune for achieving green and durable concrete [6]. To evaluate the effect of fly ash and sand dune in prepared geopolymers, we prepared seven samples with the same procedure. Compression tests were conducted using a LLoyd LR/10KN Universal Machine at room temperature and crosshead speed of 50 mm min-1for the determination of compression modulus and yield strength, according to the standard ASTM D638. Compared with geopolymers prepared by other aluminosilicates sources and under conventional method, the young's modulus and yield strength are greatly enhanced as show in Tables 7 and 8. It shows that the mecanicals properties of geopolymers depend on the content of fly ash and molar ratio fly ash /sand dune. The compression test was carried out to evaluate the compression properties of the various samples compositions in order to determine the influence of the addition of the fly ash on the compression properties of the virgin matrix. Young modulus, compressive strength and elongation at break were evaluated as a function of the mass fraction of fly ash in all series of samples. The test pieces are maintained during the test by pneumatic jaws preventing any sliding of the test piece during the traction. The initial strain rate was set at 5 mm min-1. From these data, it can be deduced that the incorporation of the fly ash into the geopolymer matrix, with different percentages, has significantly improved all of its compression properties. Thus Young's modulus increased in compositions with the highest fly ash contents, (20–50 w%) [7]. The composition of fly ash (30 w %) in geopolymer has the highest compression values. This is attributed to the interactions between the geopolymer chains and the nanometric layers of the fly ash with a decrease in the value of the Young's modulus [8]. This composition is the most compressive resistant with a maximum stress of 49.71 MPa, the most flexible (E = 2.63 GPa) and the most ductile (εr = 65.42%). This new parameter confirms the exfoliation of fly ash in geopolymers synthesized (GPs) which is in agreement with the literature [9].

## Appendix Supplementary materials

Image, application 1

Image, application 2
