# Supplementary material for: Analysis of experimental data of environmental cement prepared by fly ash of eggs shell and sand dune for reduction of carbon dioxid
Source: Data Brief. 2020 Mar 12;30:105407. doi: 10.1016/j.dib.2020.105407 (PMC7090328; doi:10.1016/j.dib.2020.105407)
Supplement: Supplementary file 2 [file mmc2.docx]

**Confirmation**

Dear editor : thank you very much for your collaboration

I inform you as much as the corresponding author (Abdelkader Rahmouni) as well as all the authors in the first version of the article (Abdelghani Brahimi, Mourad Meghachou, Hicham Abbad and Mohammed Belbachir) declare, that we have added the names of (Dr: Fatima Zohra Zeggai and Dr: Bachari Khaldoun) because they help us to correct this article especially (the language and the computer tool).you find E-mail address of authors and address of research center:

*^3^ Centre de Recherche Scientifique et Technique en Analyses Physico-chimiques (CRAPC), BP 38Bou-Ismail-RP 42004, Tipaza, Algeria.*

Dr: Fatima Zohra Zeggai: [Zeggai@yahoo.fr](mailto:Zeggai@yahoo.fr)

Dr: Bachari Khaldoun	: [bachari@yahoo.fr](mailto:bachari@yahoo.fr)

Thank you for your collaboration
